# Changes to the dynamic nature of hemagglutinin and the emergence of the 2009 pandemic H1N1 influenza virus

**DOI:** 10.1038/srep12828

**Published:** 2015-08-13

**Authors:** Sun-Woo Yoon, Noam Chen, Mariette F. Ducatez, Ryan McBride, Subrata Barman, Thomas P. Fabrizio, Robert G. Webster, Turkan Haliloglu, James C. Paulson, Charles J. Russell, Tomer Hertz, Nir Ben-Tal, Richard J. Webby

**Affiliations:** 1 Division of Virology, Department of Infectious Diseases, St. Jude Children’s Research Hospital, Memphis, TN 38105, USA; 2 Viral Infectious Disease Research Center, Korea Research Institute of Bioscience and Biotechnology, Daejeon 305-806, South Korea; 3 Department of Biochemistry and Molecular Biology, The George S. Wise Faculty of Life Sciences, Tel Aviv University, Tel-Aviv 69978, Israel; 4 INRA, UMR1225, IHAP, F-31076 Toulouse, France; 5 Department of Chemical Physiology, The Scripps Research Institute, La Jolla, CA 92037, USA; 6 Polymer Research Center and Chemical Engineering Department, Bogazici University, Bebek, Istanbul 34470, Turkey; 7 Vaccine and Infectious Disease Division, Fred Hutchinson Cancer Research Center, Seattle, WA 98109, USA

## Abstract

The virologic factors that limit the transmission of swine influenza viruses between humans are unresolved. While it has been shown that acquisition of the neuraminidase (NA) and matrix (M) gene segments from a Eurasian-lineage swine virus was required for airborne transmission of the 2009 pandemic H1N1 virus (H1N1pdm09), we show here that an arginine to lysine change in the hemagglutinin (HA) was also necessary. This change at position 149 was distal to the receptor binding site but affected virus-receptor affinity and HA dynamics, allowing the virus to replicate more efficiently in nasal turbinate epithelium and subsequently transmit between ferrets. Receptor affinity should be considered as a factor limiting swine virus spread in humans.

Despite the sporadic detection of triple reassortant swine (TRsw) H1N1 influenza viruses in humans, it was not until the 2009 pandemic, after they had obtained the NA and M gene segments from a Eurasian avian-like (EA) swine virus that they spread efficiently among humans. The molecular determinants of influenza virus transmissibility are still poorly understood despite recent advances detailing the mechanisms required for avian influenza viruses to adapt to airborne transmission in ferrets^1^,^2^,^3^,^4^. In these systems, development of airborne transmission of avian influenza viruses in ferrets coincides with a switch in receptor preference from the typical avian-virus specificity of α2,3 linked terminal sialic acids (SA) to the mammalian-virus specificity of α2,6 linked terminal SA. Considering these findings, it is somewhat perplexing that many swine-adapted influenza viruses that already possess α2,6-SA specificity are unable to efficiently transmit between ferrets and humans^5^,^6^, demonstrating that more subtle phenotypes limit the spread of these viruses in the human population.

Through analysis of HA sequences from H1N1pdm09 and swine H1 viruses we have previously identified amino acid residues that segregate with viral lineage. We were able to show that some of these residues impacted viral virulence in mice^7^. We therefore hypothesized that some of these residues may also impact viral transmission.

Here we demonstrate that the single amino acid substitution, HA R149K, was able to enhance the binding of a TRsw H1N1 virus to α2,6-SA resulting in enhanced virus replication *in vitro* and increased contact transmission in ferrets. Addition of the H1N1pdm09 NA and M gene segments to the mutant virus resulted in ferret droplet transmission to a level similar to that of the H1N1pdm09 virus itself. These results demonstrate that specific and subtle changes in the HA of H1N1pdm09 virus or its direct TRsw precursors were important factors in its emergence.

## Results

### Transmissibility of a TRsw H1N1 Virus in the Ferret Model

**Figure 1:**
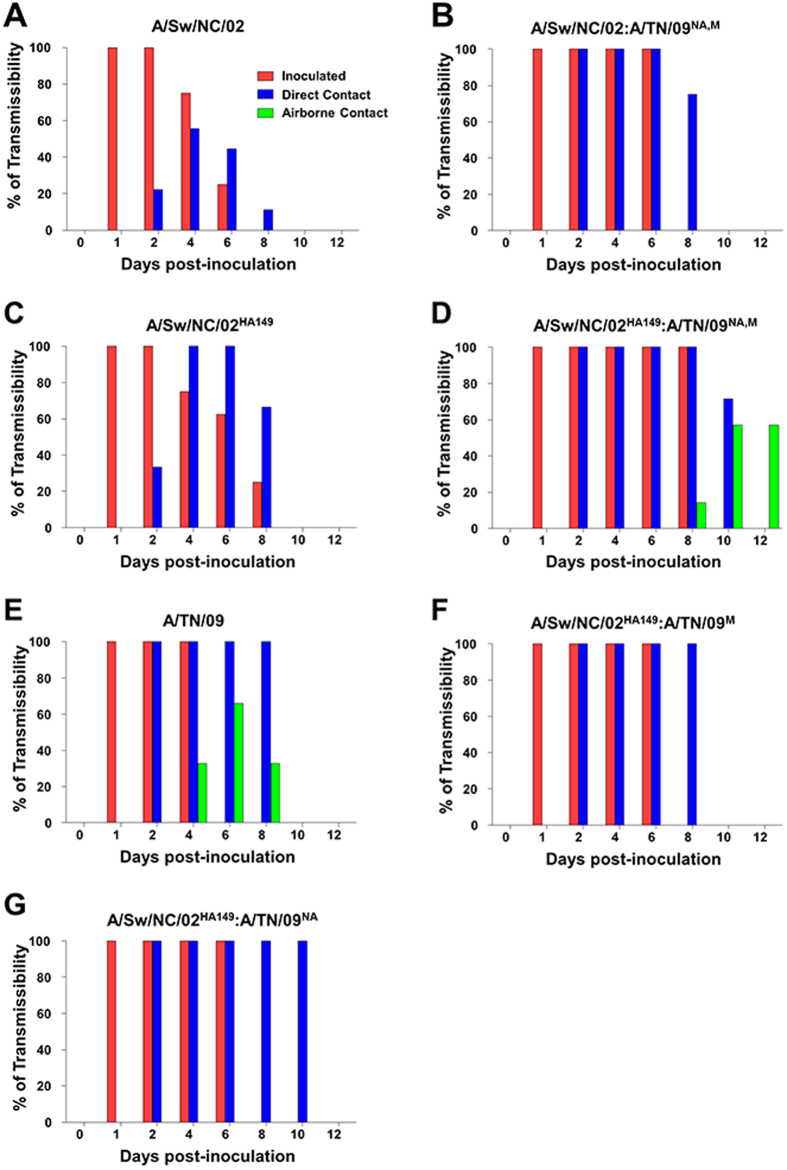
Transmissibility of the recombinant TRsw viruses in ferrets. Ferrets were inoculated intranasally with 10^6^ EID_50_/ml of NC/02 (**a**) NC/02:TN/09^NA,M^ (**b**) NC/02^HA149^ (**c**) NC/02^HA149^:TN/09^NA,M^ (**d**) TN/09 (**e**) NC/02^HA149^:TN/09^M^ (**f**) and NC/02^HA149^:TN/09^NA^ (**g**) influenza viruses. The transmissibility percentage was based on the TCID_50_ assay using nasal wash samples in each group.

In 2009, the H1N1pdm09 virus caused an influenza pandemic. Sequence analyses revealed that the H1N1pdm09 virus possessed gene segments from EA (NA and M) and TRsw swine virus lineages^8^,^9^. Two subsequent reports highlighted the importance of the NA and M segments for efficient airborne transmission of the virus in ferrets^10^,^11^. It was unexpected, therefore, that we observed increased direct contact transmission (infecting 9/9 direct contacts), but not airborne transmission (infecting 0/9 airborne contacts) when we introduced the NA and M segments from the H1N1pdm09 virus, A/Tennessee/1-560/2009 (TN/09), into A/swine/North Carolina/18161/2002 (NC/02), a TRsw H1 virus (Fig. 1a,b, Supplementary Fig. S1A and B).

We have previously described an approach to elucidate mutations that may contribute to influenza virus phenotypes^7^. We applied this approach to compare swine H1N1 to H1N1pdm09 sequences, limiting our analysis to the HA receptor binding domain (RBD) based on our hypothesis that this domain is critical to host specificity. We identified two mutations (R149K and R133_A_K; H3 numbering) that when introduced into the HA of NC/02, altered virus binding to erythrocytes and enhanced viral pathogenicity in mice^7^. Based on the fact that the 149K and 133_A_K mutations altered HA interactions with its receptor and that they were overrepresented in H1N1pdm09 viruses and underrepresented in swine H1 viruses we hypothesized that they may also have an impact on viral transmission. Indeed, introduction of the R149K mutation into the HA of NC/02 (NC/02^HA149^) led to an increase in transmission to direct contact ferrets from 5/9 for the wild type NC/02 to 9/9 for the mutant virus (*p* = 0.014) (Fig. 1c, Supplementary Fig. S1C); the R133_A_K mutation had no impact on transmission and neither mutant was detected in airborne contact animals (Fig. 1c, Supplementary Fig. S2). Viral titers were measured in the respiratory tract of both NC/02 and NC/02^HA149^ infected and contact animals on day 5 post infection to look for markers that may help explain the differences in transmission. The only difference detected was a higher titer of virus in the nasal turbinates of NC/02^HA149^ infected and contact animals (*p* = 0.035) (Table 1). Consistent with this result, NC/02^HA149^ bound more extensively to fixed ferret turbinate tissue as compared to NC/02 (Supplementary Fig. S3).

We next sought to determine if the HA R149K substitution in combination with the EA NA and M genes, as is naturally present in the H1N1pdm09 viruses, would impart airborne transmission. We replaced the NC/02^HA149^ NA and M segments with those of TN/09 (creating the virus NC/02^HA149^:TN/09^NA,M^). NC/02^HA149^:TN/09^NA,M^ was detected in 7/7 and in 4/7 animals in direct and airborne contact with intranasally infected animals, respectively, (Fig. 1d); a transmission rate similar to TN/09 itself when assayed under the same conditions (Fig. 1e)^5^,^12^. Although the percentage of contact animals becoming infected were similar between NC/02^HA149^:TN/09^NA,M^ and TN/09, the transmission of the former virus to airborne contacts was delayed, suggesting that other changes might also be important for optimal transmission. Consistent with this possibility, reversion of position 149 from K to R in TN/09 (TN/09^HA149^) did not abolish airborne transmission, but delayed time to transmission (Fig. S4). There are a number of additional differences between NC/02 and TN/09 in the vicinity of the 149 pocket, and it is possible that these may also impact transmission phenotypes.

Both the EA NA and M segments were required for the airborne transmission phenotype (Fig. 1f,g). To evaluate the activity of the NA, we measured NA enzymatic kinetics using 4-MU-NANA as a fluorogenic substrate. With 4-MU-NANA, the NA from TN/09 had significantly higher enzyme activity (a higher *V*_*MAX*_) than did the NA from NC/02 (*p* < 0.05, Supplementary Fig. S5) supporting the importance of HA:NA balance for transmission of H1N1pdm09 viruses as proposed by Yen *et al.*^10^. Overall, the EA NA and M and lysine at position 149 were all necessary, but none alone sufficient, to impart airborne transmission to NC/02.

### Receptor Binding and Replication Kinetics of NC/02^HA149^

**Figure 2:**
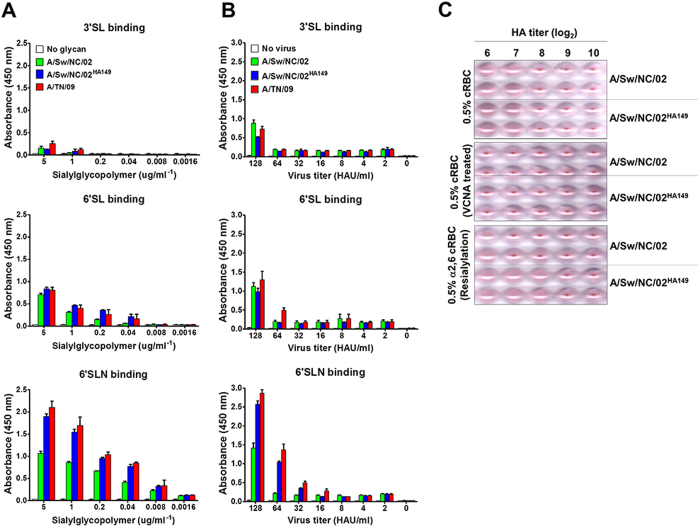
Receptor binding avidity of NC/02, NC/02^HA149^, and TN/09 influenza viruses. Each virus binding to sialylglycopolymers containing either α2,3- or α2,6-sialylated was measured by solid-phase direct binding assays (**a**) and dose-dependent direct glycan binding assays (**b**). Avidity assays were confirmed by performing a standard hemagglutinin assay with the resialylated cRBCs; untreated cRBCs (upper), VCNA-treated cRBCs (middle), or α2,6-resialylated cRBCs (bottom) (**c**). The images are representatives from four independent experiments. In each assay described NC/02^HA149^ bound more strongly to the α2,6 receptor.

We next sought to determine the mechanism for the increased transmission imparted by the HA R149K substitution. R149 is remote from the receptor binding site (Supplementary Fig. S6) and, correspondingly, we were unable to detect substantial differences in the specificity of HA receptor binding of NC/02 and NC/02^HA149^ as measured by glycan arrays (Supplementary Fig. S7). As compared to NC/02, NC/02^HA149^ did, however, have an increased binding affinity to a long α2,6 glycan as measured by solid-phase binding assays (Fig. 2a), a dose-dependent glycan binding assay (Fig. 2b), and binding to α2,6-resialylated cRBCs (Fig. 2c). Taken together, our results demonstrate that the single amino acid 149K in the background of NC/02 enhances binding avidity for α2,6-SA linkages to a level similar to that seen in H1N1pdm09-like viruses.

**Figure 3:**
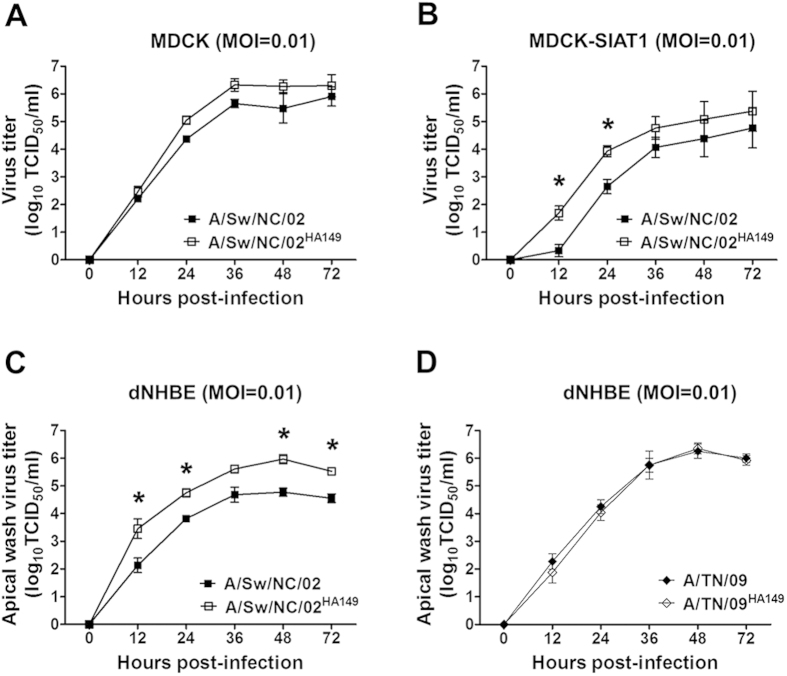
Growth of influenza viruses *in vitro*. Replication of recombinant viruses in MDCK (**a**) MDCK-SIAT1 (**b**) and dNHBE cells (**c**,**d**). Cells were infected with viruses at 0.01 MOI, and infected cultures were collected at the indicated time points. The detection limit was one log_10_TCID_50_/ml. Data are shown as means ± SD from three or four independent experiments. *p < 0.01 compared with the value for NC/02 virus.

To assess whether the HA R149K substitution had an impact on viral growth, we measured the replication efficiency of NC/02 and NC/02^HA149^ in MDCK, MDCK-SIAT1, and differentiated normal human bronchial epithelial cells (dNHBE). Both viruses grew to similar titers in MDCK cells (Fig. 3a). In contrast, NC/02^HA149^ replicated to significantly higher titers than did NC/02 in MDCK-SIAT1 cells^13^, which overexpress α2,6-SA-linked receptors (Fig. 3b). Similarly, NC/02^HA149^ grew to higher titers in dNHBE cells than did NC/02 (Fig. 3c). Consistent with the observed increased affinity to α2,6-SA-linked receptors, these findings suggest that the HA R149K substitution enhances virus replication in cells expressing high levels of α2,6-SA-linked receptors. Of note, and similar to its impact on transmission in ferrets, the reverse K149R mutation in TN/09 did not affect growth in dNHBE cells consistent with our previous data showing the mutation did not impact TN/09 binding to erythrocytes^7^.

### Position 149 is in a highly conserved cavity

**Figure 4:**
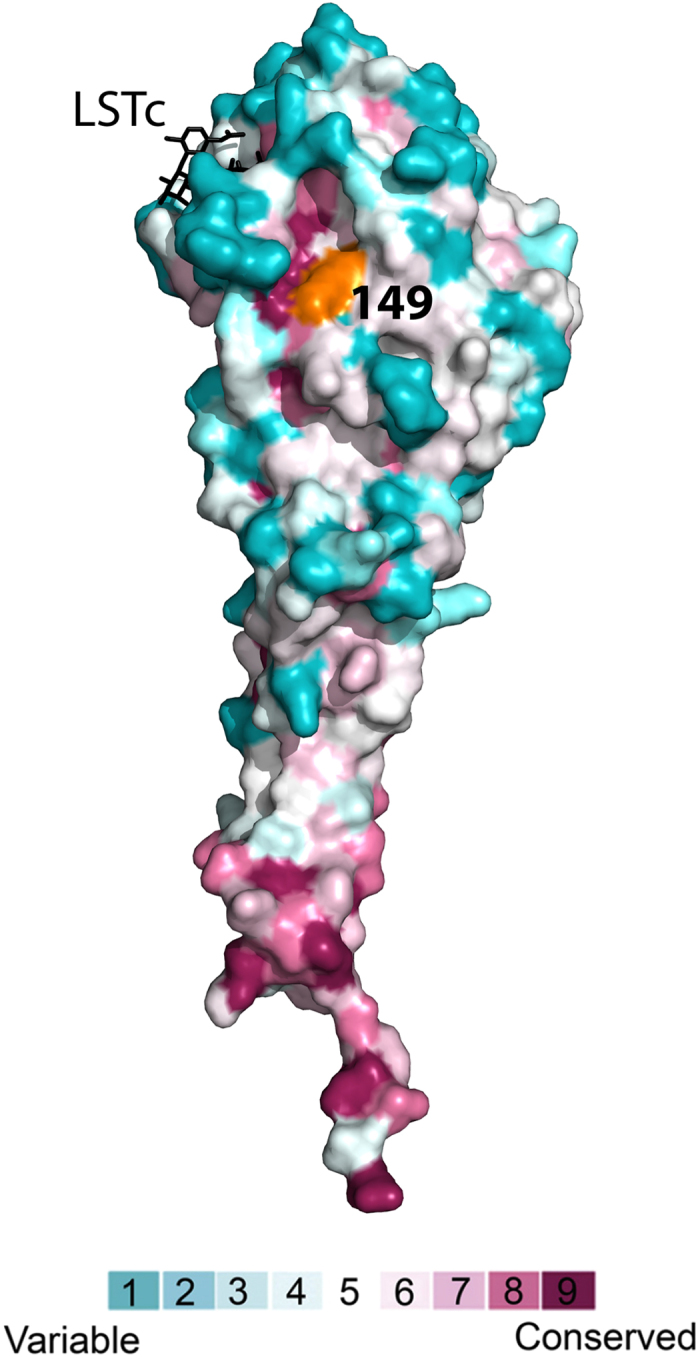
Position 149 is located in a deep and highly-conserved cleft. ConSurf analysis of the HA of A/California/04/2009 (PDB ID: 3UBE, chain E); the structure is colored by the evolutionary conservation grades using the color-coding bar, with variable-through-conserved corresponding to cyan-through-maroon. Position 149 (which is mildly conserved) is in orange. The analogue of the α2,6 host cell receptor (LSTc) is shown in black bond-sticks representation. The figure was made using PyMOL (The PyMOL Molecular Graphics System, Version 1.5 Schrödinger, LLC).

To examine the importance of position 149 for HA function we conducted evolutionary conservation analysis using ConSurf^14^,^15^. The analysis revealed that position 149 is located in a highly conserved cavity, comprised of positions 72, 74, 76, 146 and 147 (Fig. 4). The evolutionary conservation of the cavity is comparable to that of the sialic acid binding site, suggesting that it is equally important for HA function.

### Analysis of NC/02^HA149^ HA Dynamics

To help address how the 149 change may alter HA function, we conducted normal mode analysis of various H1N1 HA structures, using the Gaussian and anisotropic network models^16^,^17^,^18^, looking closely at the ten slowest modes which dominate the motion. We studied the HA1 subunit, which includes the RBD, alone and within the context of the intact HA monomer.

#### Gaussian Network Model (GNM) analysis

**Figure 5:**
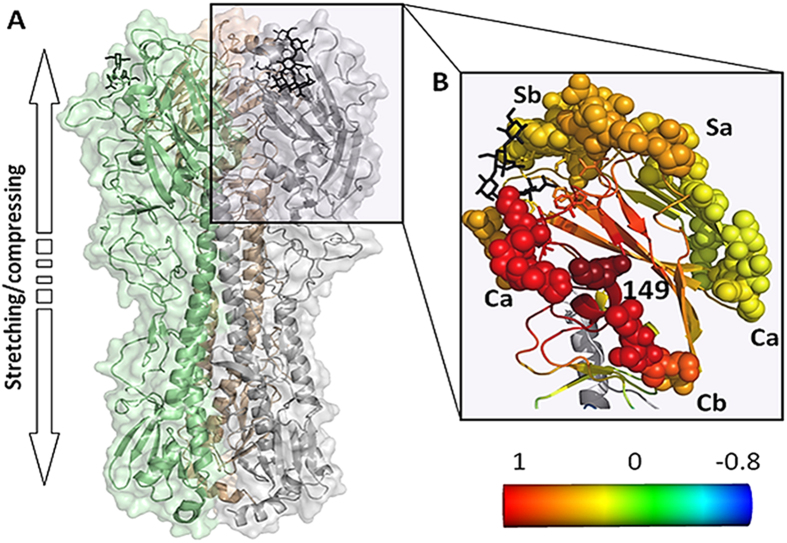
Position HA149 is a central hinge in the RBD, displaying correlated dynamic fluctuations with antigenic sites and receptor binding site residues. An overview of the HA trimer from A/California/04/2009 structure [Protein Data Bank (PDB) accession number 3UBE] in cartoon representation with each subunit in a different color. The RBDs, mediating the binding to the host cell, are at the top and the sialic acid receptors are displayed using stick model representation. Normal mode analysis revealed spring motion along the arrow, considered to be important here (**a**). Correlation between the dynamic fluctuations of position 149 and the other RBD residues, color-coded with red-through-blue corresponding to the most positively-through-most negatively cooperative motions (**b**). Amino acids of known antigenic sites are presented as spheres, and the known sialic acid-binding residues are marked with sticks; binding site residues that are also a part of an antigenic site are presented as spheres. The analogue of the α2,6 host cell receptor is shown in black bond-sticks representation. The figure was made using PyMOL (The PyMOL Molecular Graphics System, Version 1.5 Schrödinger, LLC).

We applied GNM^16^,^17^ to one of the HA1 chains of the HA protein from the H1N1pdm09 virus A/California/04/2009 (PDB ID: 3UBE)^19^,^20^. In GNM, the slowest modes describe the major fluctuations; the most cooperative motions taking place in the largest time scales. It can be seen that the stretch of positions 136–152, 173–182, and 231–240 of the A/California/04/2009 RBD fluctuate to a lesser extent than their surroundings, and serve as local hinges for the first slow mode and as major hinges in the second and especially the third slow modes (Supplementary Fig. S8). These regions are a part of the Ca antigenic and sialic acid binding sites^21^. The average correlation of the motion of position 149 with the motion of the rest of the HA1 chain is displayed on the structure of 3UBE (Fig. 5). Position 149 shows cooperative fluctuations with most of the receptor-binding and vestigial-esterase domains, especially with positions 63–88 (including the Cb antigenic region), 92–99 (a segment containing the Tyr98 binding residue), 133–153 (a part of the Ca antigenic region, containing binding site residues Lys133_A_, Gly134, Val135, Thr136, Ala137, and Trp153) and 252–256. In attempt to understand the impact of the R149K mutation on the cooperativity between residue motion in the RBD we applied a GNM cross correlation analysis to six different H1N1 variants: A/Darwin/2001/2009 (PDB ID: 3M6S)^22^, A/California/04/2009 (PDB ID: 3UBE), A/Solomon Islands/03/2006 (PDB ID: 3SM5, A/Berlin/6/2006 for HA2)^23^, A/Swine/Iowa/15/30 (PDB ID: 1RVT)^24^, A/South Carolina/1/18 (PDB ID: 1RUZ)^24^, and A/Puerto Rico/8/34 (PDB ID: 1RU7)^24^; the former three have a lysine in position 149 while the latter three contain an arginine. The GNM cross correlation analysis revealed that the lysine-variants display increased motion-cooperativity between positions 134–145 providing an explanation for the impact of the R149K change on receptor binding.

**Figure 6:**
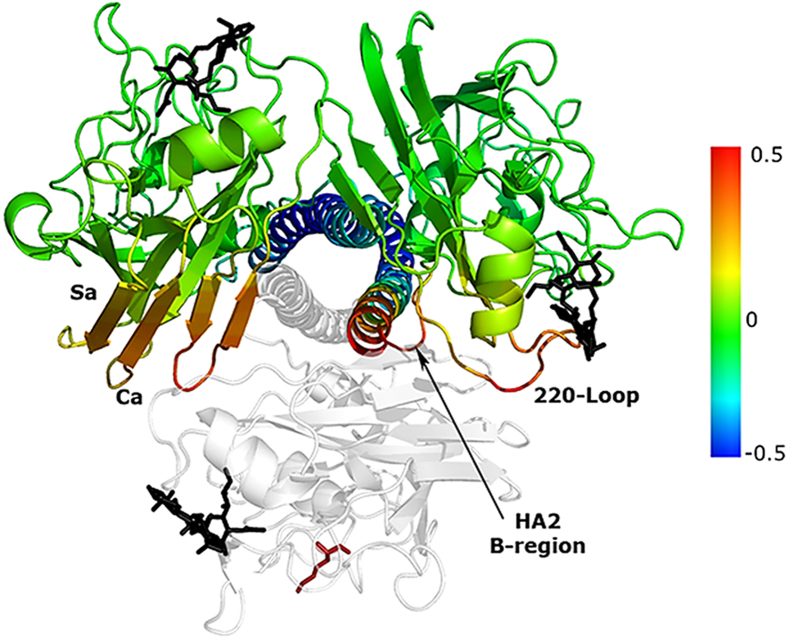
The motion in position 149 correlates with the motion of key regions of the two other monomers in the trimer. A top-down view of the HA trimer from the human A/California/04/2009 H1N1 strain (PDB ID: 3UBE) in cartoon representation. The correlation between the fluctuations of position 149 (red bond-sticks model representation) of the bottom monomer (grey) and the two top monomers, color-coded with red-through-blue corresponding to the most positively-through-most negatively cooperative residue motions. Correlated fluctuations were observed with amino acids in the B-region of HA2, the sialic acid binding site (220-loop), and parts of the Ca and Sa antigenic sites. The analogue of the α2,6 host cell receptor is shown in black bond-sticks representation. The figure was made using PyMOL (The PyMOL Molecular Graphics System, Version 1.5 Schrödinger, LLC).

To better understand the role of position 149 in HA dynamics we expanded the normal mode analysis to the trimeric HA structure. We calculated the average correlations between the motions of position 149 in one of the HA monomers with motions in the other two (Fig. 6). Interestingly, position 149 shows positively correlated motion with key functional regions: it shows high positive correlation with parts of the Ca and Sa antigenic sites of one monomer and with positions 216–229 (of HA1) and 70–83 (of HA2) of the other monomer. Positions 216–229 encompass the 220-loop, including residues 226 and 228 involved in binding the sialic acid receptor. Positions 70–83 in HA2 include a part of the C-region helix (positions 76–105) and the upper part of the B-region loop (positions 55–75)^25^. The B-region undergoes conformational change at low (endosomal) pH, triggering the fusion of the viral and endosome membranes and the injection of the viral RNA into the host cell. The dynamic correlations across subunits are in keeping with the effect of the mutation on receptor binding avidity without changing the affinity.

#### Anisotropic Network Model (ANM) analysis

ANM^18^ analysis of the full HA trimer was used to reveal the 3D characteristics of its motions. HA1 fluctuations in the ten slowest modes were very similar to the average GNM fluctuations over the ten slowest modes. Reassuringly, position 149 was observed as a hinge in each of the ten modes (Supplementary Fig. S9). We next characterized the motion of the HA trimer by analyzing the cooperative motions that each of the ten slowest ANM modes describes. Three of the ten slowest modes, i.e., modes 1, 4, and 7, were non-degenerate and invariant with respect to the rotations around the trimer’s symmetry axis and should be important for symmetry-preserving motions. Position 149 appeared as a hinge in all three modes; a local hinge in mode 1 and global hinge in modes 4 and 7. Mode 1 displayed a rotational motion of the RBD’s β-sheet, whereas the stalk region appeared to rotate in the opposite direction. Mode 4 was a squeezing motion that seemed mostly important for the stalk region, causing a tightening of the trimer. Mode 7 showed a very interesting motion of vertical extraction/contraction, using the stalk region as a spring while the head region was stretched in an upward motion (Fig. 5a, Supplementary video S1). We next sought to determine if the R149K substitution is predicted to affect any of the above motions by comparing the ANM mean squared fluctuations of the three 149R and three 149K H1N1 HA’s described above. The different variants display very similar RBD fluctuations in mode 1, whereas in modes 4 and 7 there are noticeable differences between the lysine- vs. arginine-variants. Mode 7 was affected to the largest extent by the R149K mutation, with differently fluctuating residues throughout the RBD (Supplementary Fig. S10A). The most differently fluctuating residues were located in a large region comprising positions 177–179 and 199–216, which fluctuate to a lesser extent in lysine-containing HA’s (Supplementary Fig. S10B).

Differences between the HA149 lysine- and arginine-containing variants in mode 7 were even more pronounced when viewed over the entire trimer. In particular, positions 44–57 and 268–297 fluctuated to a much greater extent in the lysine-variants. Different fluctuations were also seen in the HA2 subunit in the lower part of the stalk helices at positions 37–49 and 103–122. The HA2 regions fluctuated less in the 149K variants (Supplementary Fig. S10C). The regions predicted to be affected the most from the R149K mutation were located in the F` sub-domain, implying a potential effect on a fusion-related process, but our experiments indicated that the arginine-to-lysine mutation did not affect the pH of fusion activation. Taken together, position 149 appeared as an important hinge in the ten slowest ANM modes, providing further indication for the importance of this position for HA dynamics.

## Discussion

In this study we evaluated the contribution of an HA R149K substitution to the biological function of a representative TRsw virus, NC/02. NC/02, like many swine influenza viruses of this genetic lineage, is unable to transmit between ferrets despite the presence of α2,6-SA receptor specificity^5^. The further inability of this virus to transmit in ferrets even after acquisition of the H1N1pdm09 NA and M gene segments, in so doing creating the pandemic virus’ genotype, led us to explore additional genetic changes that likely accompanied the emergence of the pandemic virus from its swine virus precursors. We had previously identified the HA R149K change amongst a panel of other substitutions that were more common in the pandemic virus lineage than in swine influenza viruses but we had not determined its impact on transmission. In the current study we were able to show that compared with the parental virus, NC/02^HA149^ had increased binding avidity to α2,6-SA, and it replicated to higher titers of infectious virus in dNHBE cells and ferret nasal turbinates. Notably, the HA R149K substitution significantly increased contact transmission efficiency between ferrets. NC/02 and NC/02^HA149^ replicated to similar levels in the trachea and lower respiratory tract of ferrets on day 5 p.i. whereas NC/02^HA149^ replicated to significantly higher titers in the nasal turbinate which has a predominance of α2,6-SA receptors^26^.

Despite the increased direct-contact transmissibility of NC/02^HA149^, it was not airborne transmissible, again consistent with the previously demonstrated lack of airborne transmissibility of North American TRsw and Eurasian swine influenza viruses in a ferret model^11^,^27^. Also consistent with previous studies^10^,^11^ we found that addition of the 2009 pandemic virus’ NA and M to NC/02^HA149^ allowed for airborne transmission. Both NA and M segments were required for this transmission. The importance of the H1N1pdm09 NA gene segment may be explained by its higher sialidase activity^10^,^11^ but the role of the M segment in transmission is unclear. While we were able to show that the HA R149K substitution and the EA M and NA segments were all required, but none alone sufficient, for the transmission phenotype of NC/02^HA149^:TN/09^NA,M^, introducing the HA K149R mutation into TN/09 did not entirely abolish airborne transmission although it impacted transmission kinetics. This, along with the fact that NC/02^HA149^:TN/09^NA,M^ had delayed transmission kinetics in comparison to TN/09 suggest that there are additional changes in TN/09 that are required for its optimal transmission.

The HA R149K mutation accounts for an increased binding affinity for the α2,6-SA receptor, increased virus replication in α2,6 containing cells, and increased transmission of the TRsw H1N1 virus. Receptor binding affinity has been implicated in a number of influenza virus phenotypes. There has been a gradual decrease of the affinity of the human H3N2 influenza viruses for their receptor since their emergence in humans in 1968^28^. In this instance the affinity changes have been associated with mutations in the 220-loop component of the RBD. A role for virus-receptor affinity in evading host immunity has also been realized whereby an enhanced binding allows escape from neutralizing antibodies^29^. Subsequent passaging of these viruses in the absence of antibody pressure led to compensatory mutations that reduced virus affinity. In our studies the 149 mutation affected affinity from a cleft distant from the receptor binding pocket. de Vries and colleagues have also observed an affinity difference between swine and human influenza viruses due to changes in HA positions 200 and 227 that are not in the receptor binding pocket itself. In this case the changes at these residues led to the loss of a potential hydrogen bond with parts of the RBD associated 190 loop^30^. Similar observations were made by Hensley and colleagues^29^.

Although distal to the receptor binding pocket, HA residue 149 forms an extensive network of salt bridges, linking a loop proximal to the receptor binding pocket to the vestigial esterase domain, which is located atop and stabilizes the membrane-proximal stalk domain (Supplementary Fig. S5). The arginine-to-lysine mutation could affect one or more of these functional domains and, indeed, in our normal mode analysis position 149 consistently appeared as a hinge-point in nearly all slow modes of motion. The analysis showed that position 149 is a part of a major dynamic network that includes the sialic acid binding and antigenic sites. As such, the R149K mutation could affect the RBD dynamics and allosterically modify characteristics of the sialic acid binding site, providing a possible explanation for why a relatively mild substitution from arginine to lysine in this position causes dramatic phenotypic changes including enhanced transmission. Position 149 is in an evolutionarily conserved cavity, perhaps a new binding pocket, which is allosterically coupled to key functional regions in the protein, including the sialic acid binding and antigenic sites. It is noteworthy that HA’s of other subtypes share similar dynamic behavior and allosteric coupling. In particular, the equivalents of position 149 in HA proteins from H5N1 (PDB ID: 2FK0) and H7N7 (4DJ6) are major hinges which are dynamically coupled to the same sialic acid binding and antigenic sites (data not shown). While this conservation of potential function across subtypes is reassuring, our computational predictions would benefit from validation through complex techniques such as scattering experiments or DXMS (Deuterium exchange mass spectrometry) proteomics. Indeed, further exploration will be needed to investigate these speculations, as well as to reveal the evolutionary advantage(s) for the virus to develop such allostery. Regardless, our calculations suggest that position 149 is a “dynamic hub” of the HA trimer and a part of the network that mediates critical functional aspects of HA activity.

Together our data suggest a model where the R149K substitution leads to changes in HA dynamics which results in an increased affinity of the HA trimer to the α2,6-SA receptor. The functional consequence of this for the virus is an ability to bind and replicate to a greater extent in the extremities of the ferret respiratory tract. While this change in anatomical replication enhances contact transmission, it is not until the more avid binding is balanced by the more active sialidase activity of the pandemic virus’ NA that airborne transmission is achieved. The weakness in this model is our inability to provide a mechanistic role for the pandemic M gene segment. It is possible that it plays a role in virus morphology which has been suggested to be an important factor for transmission of the H1N1pdm09 viruses^11^.

Our studies show the power of a combined computational and experimental approach to identify residues involved in influenza virus biological processes. The distal nature of the 149 residue as compared to the RBD would have made this an unlikely position for study based on biological predictions alone. Our finding has implications for swine H1N1 influenza viruses (including H1N1dpm09) only at this stage and further studies are warranted to assess the role of HA residue 149 in other H1N1 and other influenza virus subtypes. Of the 1254 North American swine H1 HA sequences (from 1930 to 2008) in the influenza sequence database (www.fludb.org), only 12 contained lysine at position 149, the large majority of sequences harboring arginine and only 5 sequences a non-R and non-K residue at position 149. Consistent with the finding of Jayaraman *et al.*^31^, who have shown correlations between receptor-binding affinities and experimentally enhanced H1N1pdm09 transmission, our study demonstrates that subtle changes in a residue distal to the RBD were critical for the emergence of the 2009 pandemic virus from its swine virus progenitors.

## Materials and Methods

### Viruses and cells

Recombinant reassortant viruses were generated by using DNA transfection as described previously^32^. After rescue, the full genome sequences of the recombinant viruses were verified by performing RT-PCR and sequencing analysis. Stock viruses were stored at −80 °C until use. Mutagenesis was conducted by using the QuikChange™ Site-Directed Mutagenesis Kit (Stratagene). The Madin-Darby canine kidney (MDCK) and human embryonic kidney (293T) cells were obtained from the American Type Culture Collection (Manassas, VA) and maintained as previously described. To determine multistep growth curves, we used MDCK-SIAT1^13^ and dNHBE cells^33^, which were maintained as described previously. All cells were grown at 37 °C in 5% CO_2_. A summary Table of recombinant viruses used in this study is presented as Supplementary Table 1.

### Transmission experiments in ferrets

All animal experiments were approved by the St. Jude Animal Care and Use Committee, and complied with the policies of the National Institutes of Health and the Animal Welfare Act. Male ferrets aged 3 to 5 months were purchased from Marshall Farms (North Rose, NY) and all ferrets were determined to be seronegative to circulating human influenza viruses. To study viral pathogenicity and transmissibility, we used experimental groups composed of one inoculated ferret and two naive contact ferrets. The donor ferrets were inoculated intranasally with 106 EID_50_/ml of virus in 1 ml sterile phosphate-buffered saline while under isoflurane anesthesia. Twenty-four hours p.i., a naive ferret was added to the cage with each donor ferret to test direct contact (DC) transmission, and a second naive ferret was added to the other half of the cage which was separated by double layers of wire mesh allowing only airborne contact (AC). The naive AC ferrets were handled before the naive DC ferrets were, and donor ferrets were handled last; additionally, separate gloves and tools were used for AC ferrets. To measure viral shedding, nasal washes were collected from ferrets on days 1, 2, 4, 6, 8, 10, and 12 p.i. (or days 1, 3, 5, 7, 9, and 11 post-contact). The virus titers were expressed as log_10_ TCID_50_/ml in MDCK cells. The limit of virus detection was 1 log_10_ TCID_50_/ml.

### Virus replication kinetics

The virus growth kinetics were determined by calculating the 50% tissue culture infectious dose (TCID_50_) as described previously^34^. To generate virus replication kinetics, MDCK and MDCK-SIAT1 were infected with NC/02 and NC/02^HA149^ viruses at a multiplicity of infection (MOI) of 0.01 PFU per cell. The virus inoculums were removed after 1 h. Cells were then washed and infection medium added (containing 1 μg/ml of TPCK-treated trypsin). Supernatants were collected 12, 24, 36, 48, and 72 h p.i and stored at −80 °C for titration by TCID_50_. Growth kinetics was also determined in dNHBE cells grown in an air-liquid interface model. dNHBE cells were apically inoculated with each virus at the indicated multiplicities of infection (MOI). After 1 h incubation, unbound virus was removed by aspiration and the cells washed with PBS 3 times. Viruses released apically were harvested by addition of 300 μl of 0.05% BSA-BEBM which was allowed to equilibrate at 37 °C for 30 minutes before collection. Viral titers were determined by titration on MDCK cells.

### Preparation of sialidase-treated cRBCs

All sialic acid residues were enzymatically removed from cRBCs by incubation with the 50 mU Vibrio cholera neuraminidase (VCNA; Roche) in 8 mM calcium chloride at 37 °C for 1 h and then resialylated followed by using either α2,6-(N)-sialyltransferase or α2,3-(N)-sialyltransferase (Sigma) at 37 °C for 4 h^35^. The receptor binding avidity of NC/02 or NC/02^HA149^ virus were determined by performing standard HA assays using 0.5% modified cRBCs.

### Receptor binding assays

For the binding assay, viruses were grown in eggs, purified, and concentrated over a cushion of 25% sucrose in 1 × STE (0.1 M NaCl, 10 mM Tris-HCl, 1 mM EDTA pH8.0) buffer, and ultracentrifuged at 25,000 rpm for 1 h at 4 °C. Concentrated virus titers were determined by using an HA assays with 0.5% cRBCs. Biotinylated glycans of α2,3′SL (Neu5Acα2-3Galβ1-4Glcβ-PAA-Biotin), α2,6′SL (Neu5Acα2-6Galβ1-4Glcβ-PAA-Biotin), and α2,6′SLN (Neu5Acα2-6Galβ1-4GlcNAcβ-PAA-Biotin) were purchased from GlycoTech Corporation (http://www.glycotech.com, USA). The receptor-binding capacity of viruses was confirmed by use of a solid-phase direct binding assay^36^,^37^ and dose-dependent glycan binding assay^38^ as previously described. For solid-phase direct binding assay, 96-well microtitre plates (Nunc) were incubated with 10 μg/ml of fetuin (Sigma) in PBS at 4 °C overnight. Fetuin-coated plates were blocked with 0.2 ml of PBS containing 5% BSA at room temperature for 1 h. After four washes with ice-cold PBS, the plates were incubated with the influenza virus (32 HAU/ml) at 4 °C overnight. After washing as described above, 0.1 ml of different concentrations of biotinylated glycans was added to each well of the plates. After 2 h incubation at 4 °C, the plates were washed three times with ice-cold PBS and then incubated with 0.1 ml of horseradish peroxidase (HRP)-conjugated streptavidin (1000-fold diluted in PBS; Invitrogen) at 4 °C. After washing, the plates were incubated with 0.05 ml of TMB substrate (Sigma) for 10 min at room temperature, the reaction was stopped with 0.05 ml of 50 mM HCl, and then optical density at 450 nm was measured in a Synergy 2 multi-mode microplate reader (BioTek Instruments). For the dose-dependent virus binding assay, streptavidin-coated 384-well microplates (Pierce) were loaded to the full capacity of each well by incubating the well with 50 μl of 1 μg/ml biotinylated glycan in PBS containing 1% BSA overnight at 4 °C. After excess biotinylated glycans were washed three times with PBS with 0.05% Tween-20 (PBS-T), each of the wells was blocked with PBS containing 1% BSA for 2 h at 4 °C. Following the blocking, 50 μl of diluted virus was added to each well and incubated overnight at 4 °C. After excess virus was washed five times with ice-cold PBS, each of the wells was incubated with 100 ng of NC/02 virus-specific monoclonal antibody and incubated at 4 °C overnight. After removal of the antibody solution, the wells were incubated with the anti-mouse HRP conjugated antibody (1:1000 diluted in PBS containing 1% BSA; Sigma) for 1 h at room temperature. After seven washes with PBS-T, the binding signals were determined based on the HRP activity as described above.

### Ferret organ collection and virus titration

To determine the tropism and replication efficiency of the NC/02 and NC/02^HA149^ viruses in ferret organs, we inoculated two ferrets with 10^6^ EID_50_/ml of virus. On day 5 p.i, organs were collected from the nasal turbinate, trachea (upper and lower), lungs (5 lobes), small intestine, spleen, and liver. For the virus titration of organs, each tissue was weighed and homogenized in sterile PBS with antibiotics. Virus titers were measured in MDCK cells and expressed as TCID_50_/gram of tissue.

### Virus attachment on nasal turbinate

For virus labeling, 100 μl of purified virus was incubated with 50 μg of AlexaFluor488-amine reactive dye (Invitrogen) at 4 °C for 2 h. To remove all unbound fluorescence, labeled viruses were dialyzed against PBS (containing 1 mM EDTA) in a MWCO Slide-A-Lyzer MINI dialysis unit (Thermo Scientific) at 4 °C overnight^39^. Tissues were removed from uninfected ferrets. The Alexa488-labeled NC/02 and NC/02^HA149^ viruses were added (100 HAU/ml) and incubated at 4 °C overnight. The tissues were formalin-fixed paraffin embedded, deparaffinized with xylene, and hydrated with alcohol. To visualize the cell nuclei, sections were counterstained with DAPI (Invitrogen) and attached virus viewed under a Zeiss LSM510 laser scanning confocal microscope^40^.

### NA kinetic

NA kinetics studies used NC/02, NC/02^HA149^, NC/02^HA149^:TN/09^NA,M^, NC/02:TN/09^NA,M^, and TN/09 viruses at 1 × 10^6^ pfu/ml virus doses. Viruses were incubated with 2′-(4-Methylumbelliferyl)|-α-DN-acetylneuraminic (4-MU-NANA; Sigma) substrate (final concentration, 0 to 5000 μM) and NA kinetics were determined by fluorescence of the released 4-methylumbelliferone as measured every 30 sec for 30 min by using Synergy 2 multi-mode microplate reader with excitation and emission wavelengths of 355 and 460 nm^10^. The data analyses were fit to the Michaelis-Menten equation by nonlinear regression (Prism; GraphPad version 5.03) to determine the Michaelis constant (*KM*) and maximum velocity (*Vmax*) of substrate conversion.

### Elastic network model analysis

#### GNM

The total potential of a protein structure in the GNM^16^,^17^ is given by


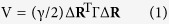


Where Δ**R** is an N-dimensional vector of the fluctuations of Δ**R**_i_ in **R**_i_ of the individual sites, Δ**R**^T^ is its transpose. Γ is the connectivity (or Kirchhoff) matrix, describing the interaction of residues within a distance cut-off (the commonly used value of 7 Å was used here) by a harmonic potential function with a force constant γ. The correlation between Δ**R**_i_ and Δ**R**_j_ is calculated as


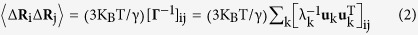


λ_k_ is the k-th eigenvalue of Γ and is representative of the frequency of the k-th mode of motion, which is (γ λ_i_)^½^. **u**_k_ is the k-th eigenvector and gives the shape of the k-th mode as a function of residue index. k_B_ is the Boltzmann constant and T is the absolute temperature in degrees Kelvin. When i = j Eq. 2 gives the self-correlations of Δ**R**_i_; i.e. mean squared residue fluctuations. This equation provides a simple means by which the dynamics can be decomposed into a series of N-1 modes for N number of interacting residues. The cooperatively moving structural units are suggested by the slowest modes and the localized fluctuations of residues are described by the fast modes.

#### ANM

ANM^18^ predicts the directionalities of the collective motions in addition to their magnitudes. New conformations of a given structure are generated by ANM to describe the fluctuations of residues from the average in the principal directions of motion. In ANM, Γ is replaced by the Hessian matrix **H** (with the commonly used distance cut-off of 18 Å). The correlation between the position fluctuations **R**_i_ and **R**_j_ of residues i and j, Δ**R**_i_ and Δ**R**_j_, decomposed into 3N-6 modes of motions is then given by


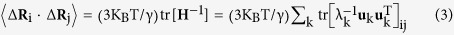


tr[**H**^−1^]_ij_ is the trace of the ij-th submatrix [**H**^−1^]_ij_ of **H**^−1^. It refers to the three different components of Δ**R**_i_ and Δ**R**_j_; whereas, when i = j, the self-correlations of the components Δ**R**_i_ are obtained. Here, with the knowledge of the fluctuation vectors, i.e. eigenvectors, we construct and view pairs of alternative conformations sampled by the individual modes. Squared fluctuations (eigenvectors) provide the residue mobilities.

### Glycan array analysis

#### Virus labeling

Inactivated viruses were directly labeled using a using a modification of a method to specifically introduce an addressable biotin tag onto terminal galactose moieties of glycoprotein glycans^41^. This method takes advantage of the fact that N-linked glycans of influenza terminate in galactose due the influenza neuraminidase. Thus, galactose oxidase can be used to introduce an aldehyde, which can be reacted with a biotin-oxyamine to tag the galactose with biotin^41^. Briefly brief, virus was incubated at 512 HAU in the presence of galactose oxidase (50 U/ml, Worthington Biochemical), aniline (30 mM, Sigma) and biotin-oxime (250 mM, Biotium) in PBS for 90 min at 37 °C. Done in ‘one pot’, the galactose oxidase introduces an aldehyde group at C6 of galactose, which in the presence of aniline efficiently reacts with the oxamine to form a stable substituted oxime bond. To remove residual enzyme and reagents, biotinylated virus is then passed over a gel filtration spin-column (Sephadex G50) to remove residual enzyme and reagents in PBS containing BSA to achieve a final concentration of 512 HAU viruses in PBS containing 3% BSA.

#### Glycan screening

Viruses were screened on a custom sialoside array, prepared as previously described^42^. Briefly, 58 amine-linkered glycans were covalently immobilized on to NHS-ester functionalized glass microscope slides (SlideH, Schott/Nexterion) using a MicroGridII robotic array printer (Digilab Global) equipped with Stealth SMP4B microarray pins (Telechem). Compounds were spotted in replicates of 6, and, following 1 h of humidification, were washed in blocking buffer (50 mM ethanolamine in 50 mM borate buffer, pH 9.2) to remove any unbound compound and quench remaining NHS-ester residues. Slides were stored dessicated before use. To assess virus binding, labeled virus was applied to the slide at a concentration of 512 HAU in PBS containing 3% BSA, and allowed to incubate for 1 h and then washed. Arrays were washed using 3 successive exchanges of PBS containing 3% BSA and 3 exchanges of PBS on the array surface. Following washing, 100 μl of streptavidin-AlexaFluor488 (2 μg/ml; Invitrogen) in PBS was applied to the array and allowed to incubate 1h and washed. Arrays were washed using 3 successive exchanges of PBS and then dipped 4 times in ddH_2_O. Washed arrays were dried by centrifugation and then scanned for Alexa488 signal on a confocal microarray scanner (ScanArray Express, Perkin Elmer). Resultant images were analyzed in Imagene (Biodiscovery) and mean signal minus background values for each spotted compound were calculated and plotted using MS Excel.

### Statistical Analysis

Statistically significant differences in virus titers among the groups were determined by one-way and two-way ANOVA followed by Tukey’s posttest using Prism software; values were considered significantly different when the *p* value was less than 0.05.

## Additional Information

**How to cite this article**: Yoon, S.-W. *et al.* Changes to the dynamic nature of hemagglutinin and the emergence of the 2009 pandemic H1N1 influenza virus. *Sci. Rep.* **5**, 12828; doi: 10.1038/srep12828 (2015).

## Supplementary Material

Supplementary Information

supplymentary video 1

## Figures and Tables

**Table 1:** Amino acid differences between NC/02 and other North American swine H1 influenza viruses from 1930 to 2008.

**Virus name**	Amino acid change*	**Accession number**	**Subtype**
A/swine/IL/00685/2005	R → N	ACM17276	H1N1
A/swine/Kentucky/SG1167/2003	R → I	AHB51196	H1N1
A/swine/Minnesota/07002083/2007	R → N	ACL79904	H1N1
A/swine/Wisconsin/464/98	R → Q	AAF87284	H1N1
A/swine/Iowa/46519-4/2008	R → Y	ADO34923	H1N1

^*^Presence of other amino acids at position 149 (H3 numbering) in 398 full-length HA sequences of pre 2009 (from 1930 to 2008) North American swine H1 influenza.

## References

[b1] Belser J. A. *et al.* Influenza virus respiratory infection and transmission following ocular inoculation in ferrets. PLoS Pathog 8, e1002569 (2012). 22396651 10.1371/journal.ppat.1002569 PMC3291616

[b2] Kimble J. B., Sorrell E., Shao H., Martin P. L. & Perez D. R. Compatibility of H9N2 avian influenza surface genes and 2009 pandemic H1N1 internal genes for transmission in the ferret model. Proc. Natl Acad. Sci. USA 108, 12084–12088 (2011). 21730147 10.1073/pnas.1108058108 PMC3141953

[b3] Imai M. *et al.* Experimental adaptation of an influenza H5 HA confers respiratory droplet transmission to a reassortant H5 HA/H1N1 virus in ferrets. Nature 486, 420–428 (2012). 22722205 10.1038/nature10831 PMC3388103

[b4] Herfst S. *et al.* Airborne transmission of influenza A/H5N1 virus between ferrets. Science 336, 1534–1541 (2012). 22723413 10.1126/science.1213362 PMC4810786

[b5] Barman S. *et al.* Pathogenicity and transmissibility of North American triple reassortant swine influenza A viruses in ferrets. PLoS Pathog. 8, e1002791 (2012). 22829764 10.1371/journal.ppat.1002791 PMC3400563

[b6] Belser J. A. *et al.* Pathogenesis and transmission of triple-reassortant swine H1N1 influenza viruses isolated before the 2009 H1N1 pandemic. J. Virol. 85, 1563–1572 (2011). 21123386 10.1128/JVI.02231-10 PMC3028905

[b7] Meroz D. *et al.* Putative amino acid determinants of the emergence of the 2009 influenza A (H1N1) virus in the human population. Proc. Natl Acad. Sci. USA 108, 13522–13527 (2011). 21808039 10.1073/pnas.1014854108 PMC3158228

[b8] Dawood F. S. *et al.* Emergence of a novel swine-origin influenza A (H1N1) virus in humans. N. Engl. J. Med. 360, 2605–2615 (2009). 19423869 10.1056/NEJMoa0903810

[b9] Smith G. J. *et al.* Origins and evolutionary genomics of the 2009 swine-origin H1N1 influenza A epidemic. Nature 459, 1122–1125 (2009). 19516283 10.1038/nature08182

[b10] Yen H. L. *et al.* Hemagglutinin-neuraminidase balance confers respiratory-droplet transmissibility of the pandemic H1N1 influenza virus in ferrets. Proc. Natl Acad. Sci. USA 108, 14264–14269 (2011). 21825167 10.1073/pnas.1111000108 PMC3161546

[b11] Lakdawala S. S. *et al.* Eurasian-origin gene segments contribute to the transmissibility, aerosol release, and morphology of the 2009 pandemic H1N1 influenza virus. PLoS Pathog. 7, e1002443 (2011). 22241979 10.1371/journal.ppat.1002443 PMC3248560

[b12] Duan S. *et al.* Oseltamivir-resistant pandemic H1N1/2009 influenza virus possesses lower transmissibility and fitness in ferrets. PLoS Pathog. 6, e1001022 (2011). 20686654 10.1371/journal.ppat.1001022 PMC2912389

[b13] Matrosovich M., Matrosovich T., Carr J., Roberts N. A. & Klenk H. D. Overexpression of the alpha-2,6-sialyltransferase in MDCK cells increases influenza virus sensitivity to neuraminidase inhibitors. J. Virol. 77, 8418–8425 (2003). 12857911 10.1128/JVI.77.15.8418-8425.2003 PMC165236

[b14] Celniker G. *et al.* ConSurf: Using Evolutionary Data to Raise Testable Hypotheses about Protein Function. Isr. J. Chem. 53, 199–206 (2013).

[b15] Glaser F. *et al.* ConSurf: identification of functional regions in proteins by surface-mapping of phylogenetic information. Bioinformatics 19, 163–164 (2003). 12499312 10.1093/bioinformatics/19.1.163

[b16] Bahar I., Atilgan A. R. & Erman B. Direct evaluation of thermal fluctuations in proteins using a single-parameter harmonic potential. Fold. Des. 2, 173–181 (1997). 9218955 10.1016/S1359-0278(97)00024-2

[b17] Haliloglu T., Bahar I. & Erman B. Gaussian dynamics of folded proteins. Physical Review Letters 79, 3090–3093 (1997).

[b18] Atilgan A. R. *et al.* Anisotropy of fluctuation dynamics of proteins with an elastic network model. Biophys. J. 80, 505–515 (2001). 11159421 10.1016/S0006-3495(01)76033-X PMC1301252

[b19] Berman H. M. *et al.* The Protein Data Bank. Nucleic Acids Res 28, 235–242 (2000). 10592235 10.1093/nar/28.1.235 PMC102472

[b20] Xu R. *et al.* Structural basis of preexisting immunity to the 2009 H1N1 pandemic influenza virus. Science 328, 357–360 (2010). 20339031 10.1126/science.1186430 PMC2897825

[b21] Caton A. J., Brownlee G. G., Yewdell J. W. & Gerhard W. The antigenic structure of the influenza virus A/PR/8/34 hemagglutinin (H1 subtype). Cell 31, 417–427 (1982). 6186384 10.1016/0092-8674(82)90135-0

[b22] Yang H., Carney P. & Stevens J. Structure and Receptor binding properties of a pandemic H1N1 virus hemagglutinin. PLoS Curr. 2, RRN1152 (2010). 10.1371/currents.RRN1152 PMC2846141 20352039

[b23] Whittle J. R. *et al.* Broadly neutralizing human antibody that recognizes the receptor-binding pocket of influenza virus hemagglutinin. Proc. Natl Acad. Sci. USA 108, 14216–14221 (2011). 21825125 10.1073/pnas.1111497108 PMC3161572

[b24] Gamblin S. J. *et al.* The structure and receptor binding properties of the 1918 influenza hemagglutinin. Science 303, 1838–1842 (2004). 14764886 10.1126/science.1093155

[b25] Bullough P. A., Hughson F. M., Skehel J. J. & Wiley D. C. Structure of influenza haemagglutinin at the pH of membrane fusion. Nature 371, 37–43 (1994). 8072525 10.1038/371037a0

[b26] Munster V. J. *et al.* Pathogenesis and transmission of swine-origin 2009 A(H1N1) influenza virus in ferrets. Science 325, 481–483 (2009). 19574348 10.1126/science.1177127 PMC4814155

[b27] Shinde V. *et al.* Triple-reassortant swine influenza A (H1) in humans in the United States, 2005-2009. N. Engl. J. Med. 360, 2616–2625 (2009). 19423871 10.1056/NEJMoa0903812

[b28] Lin Y. P. *et al.* Evolution of the receptor binding properties of the influenza A(H3N2) hemagglutinin. Proc. Natl Acad. Sci. USA 109, 21474–21479 (2012). 23236176 10.1073/pnas.1218841110 PMC3535595

[b29] Hensley S. E. *et al.* Hemagglutinin receptor binding avidity drives influenza A virus antigenic drift. Science 326, 734–736 (2009). 19900932 10.1126/science.1178258 PMC2784927

[b30] de Vries R. P. *et al.* Only two residues are responsible for the dramatic difference in receptor binding between swine and new pandemic H1 hemagglutinin. J. Biol. Chem. 286, 5868–5875 (2011). 21173148 10.1074/jbc.M110.193557 PMC3037699

[b31] Jayaraman A. *et al.* A single base-pair change in 2009 H1N1 hemagglutinin increases human receptor affinity and leads to efficient airborne viral transmission in ferrets. PloS one 6, e17616 (2011). 21407805 10.1371/journal.pone.0017616 PMC3047569

[b32] Hoffmann E., Neumann G., Kawaoka Y., Hobom G. & Webster R. G. A. DNA transfection system for generation of influenza A virus from eight plasmids. Proc. Natl Acad. Sci. USA 97, 6108–6113 (2000). 10801978 10.1073/pnas.100133697 PMC18566

[b33] Chan R. W. *et al.* Influenza H5N1 and H1N1 virus replication and innate immune responses in bronchial epithelial cells are influenced by the state of differentiation. PloS one 5, e8713 (2010). 20090947 10.1371/journal.pone.0008713 PMC2806912

[b34] Reed L. J. & Muench H. A. simple method of estimating fifty percent endpoints. Am. J. Hyg. 27, 493–497 (1938).

[b35] Glaser L. *et al.* A single amino acid substitution in 1918 influenza virus hemagglutinin changes receptor binding specificity. J. Virol. 79, 11533–11536 (2005). 16103207 10.1128/JVI.79.17.11533-11536.2005 PMC1193621

[b36] Yamada S. *et al.* Haemagglutinin mutations responsible for the binding of H5N1 influenza A viruses to human-type receptors. Nature 444, 378–382 (2006). 17108965 10.1038/nature05264

[b37] Matrosovich M. N. & Gambaryan A. S. Solid-phase assays of receptor-binding specificity. Methods Mol. Biol. 865, 71–94 (2012). 22528154 10.1007/978-1-61779-621-0_5

[b38] Pearce M. B. *et al.* Pathogenesis and transmission of swine origin A(H3N2)v influenza viruses in ferrets. Proc. Natl Acad. Sci. USA 109, 3944–3949 (2012). 22355116 10.1073/pnas.1119945109 PMC3309732

[b39] Bradley K. C. *et al.* Comparison of the receptor binding properties of contemporary swine isolates and early human pandemic H1N1 isolates (Novel 2009 H1N1). Virology 413, 169–182 (2011). 21353280 10.1016/j.virol.2011.01.027

[b40] Shinya K. *et al.* Avian flu: influenza virus receptors in the human airway. Nature 440, 435–436 (2006). 16554799 10.1038/440435a

[b41] Ramya T. N., Weerapana E., Cravatt B. F. & Paulson J. C. Glycoproteomics enabled by tagging sialic acid or galactose terminated glycans. Glycobiology 23, 211–21 (2012). 23070960 10.1093/glycob/cws144 PMC3531297

[b42] Xu R. *et al.* Functional balance of the hemagglutinin and neuraminidase activities accompanies the emergence of the 2009 H1N1 influenza pandemic. J. Virol. 86, 9221–9232 (2012). 22718832 10.1128/JVI.00697-12 PMC3416152

