# Supplementary material for: Changes to the dynamic nature of hemagglutinin and the emergence of the 2009 pandemic H1N1 influenza virus
Source: Sci Rep. 2015 Aug 13;5:12828. doi: 10.1038/srep12828 (PMC4534793; doi:10.1038/srep12828)
Supplement: Supplementary Information [file srep12828-s1.doc]

**Changes to the dynamic nature of hemagglutinin and the emergence of the 2009 pandemic H1N1 influenza virus**

Sun-Woo Yoon^1, 2^, Noam Chen^3^, Mariette F. Ducatez^1,4^, Ryan McBride^5^, Subrata Barman^1^, Thomas P. Fabrizio^1^, Robert G. Webster^1^, Turkan Haliloglu^6^, Jim C. Paulson^5^, Charles J. Russell^1^, Tomer Hertz^7^, Nir Ben-Tal^3^, Richard J. Webby^1^*

^1^ Division of Virology, Department of Infectious Diseases, St. Jude Children’s Research Hospital, Memphis, TN 38105, USA

^2^ Viral Infectious Disease Research Center, Korea Research Institute of Bioscience and Biotechnology, Daejeon 305-806, South Korea

^3^ Department of Biochemistry and Molecular Biology, The George S. Wise Faculty of Life Sciences, Tel Aviv University, Tel-Aviv 69978, Israel

^4^ INRA, UMR1225, IHAP, F-31076 Toulouse, France

^5^ Department of Chemical Physiology, The Scripps Research Institute, La Jolla, CA 92037, USA

^6^ Polymer Research Center and Chemical Engineering Department, Bogazici University, Bebek, Istanbul 34470, Turkey

^7^ Vaccine and Infectious Disease Division, Fred Hutchinson Cancer Research Center, Seattle, WA 98109, USA

**Supplementary Information**

Supplementary table 1. Characteristics of recombinant NC/02 viruses.

| Virus list* | HA mutation** | Reverse genetic backbone | Gene constellation** |
| --- | --- | --- | --- |
| NC/02 | - | NC/02 | - |
| NC/02HA^149^ | R149K | NC/02 | - |
| NC/02HA^133^ | R133AK | NC/02 | - |
| TN/09 | - | TN/09 | - |
| TN/09HA^149^ | K149R | TN/09 | - |
| NC/02:TN/09^NA,M^ | - | NC/02 | NA and M gene from TN/09 |
| NC/02HA^149^:TN/09^NA,M^ | R149K | NC/02 | NA and M gene from TN/09 |

* NC/02; A/swine/North Carolina/18161/2002, TN/09; A/Tennessee/1-560/2009

** -; no change

**Fig. S1**

**A**

**B**

**C**

**D**

**E**

**F**

**Fig. S2**

**A**

**B**

**Fig. S3**

**A/Sw/NC/02**


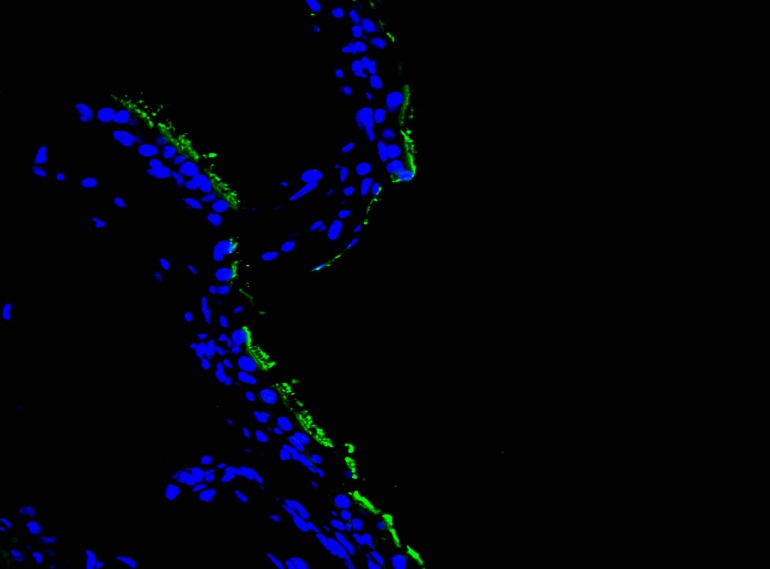

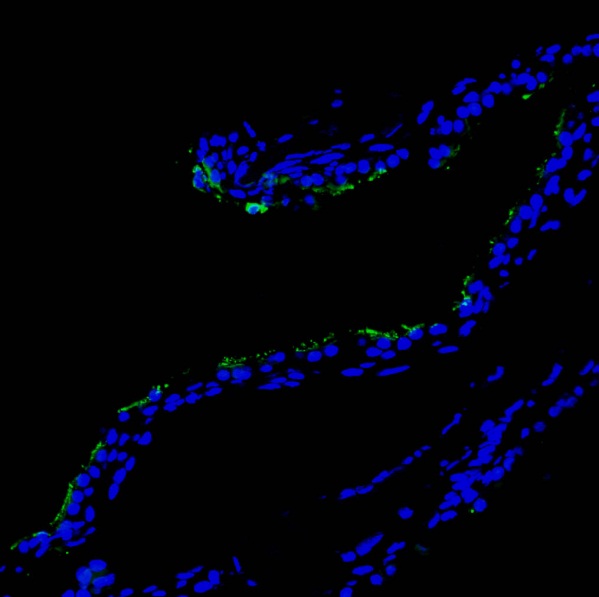


**A/Sw/NC/02^HA149^**


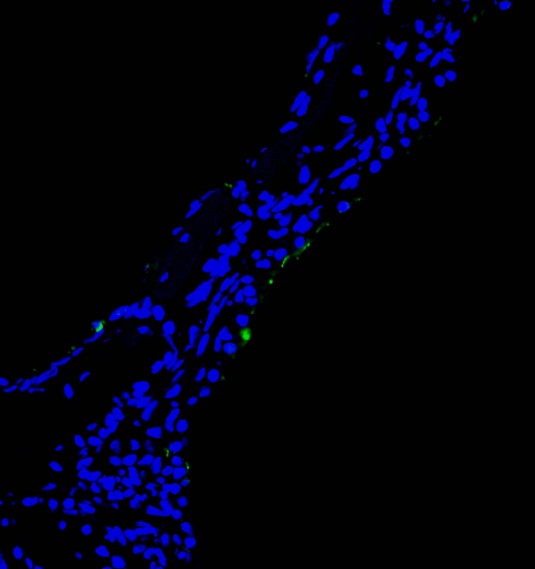


**No virus**

**A**

**B**

**C**

**Fig. S4**

**
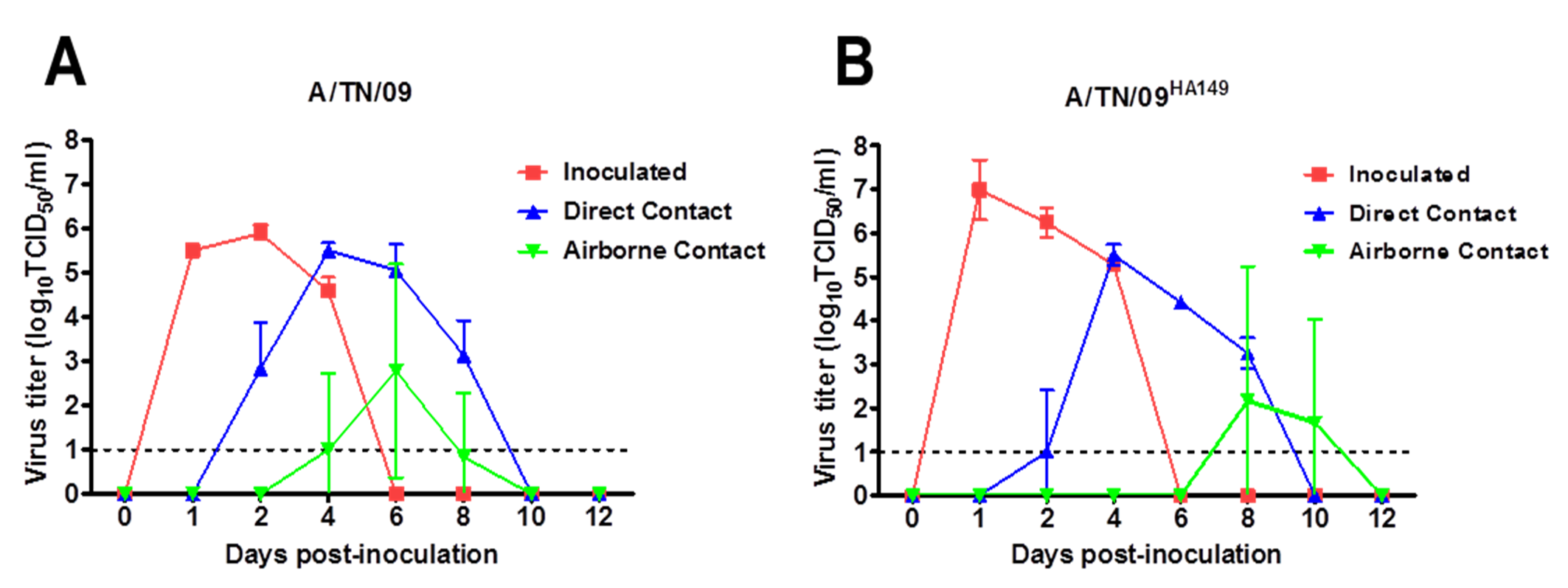
**

**Fig. S5**


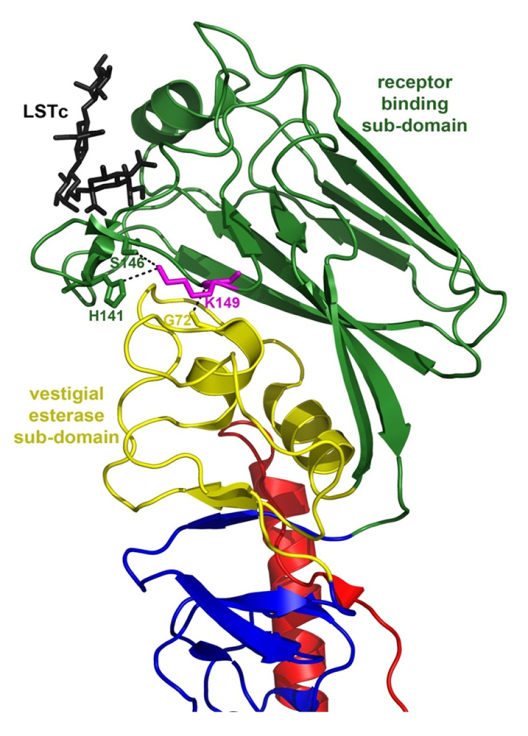
**Fig. S6**

**Fig. S7**

**A**

**B**


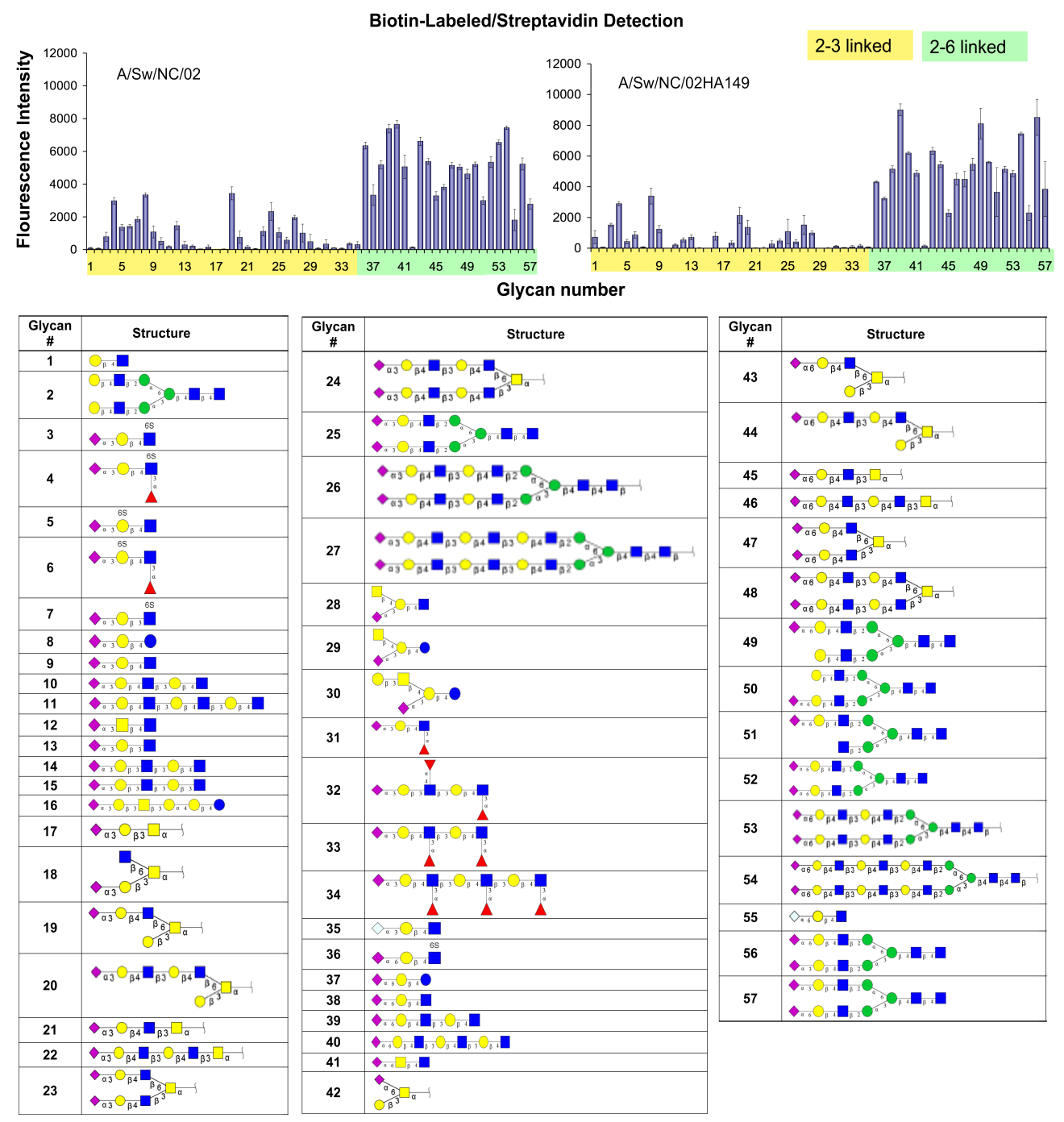


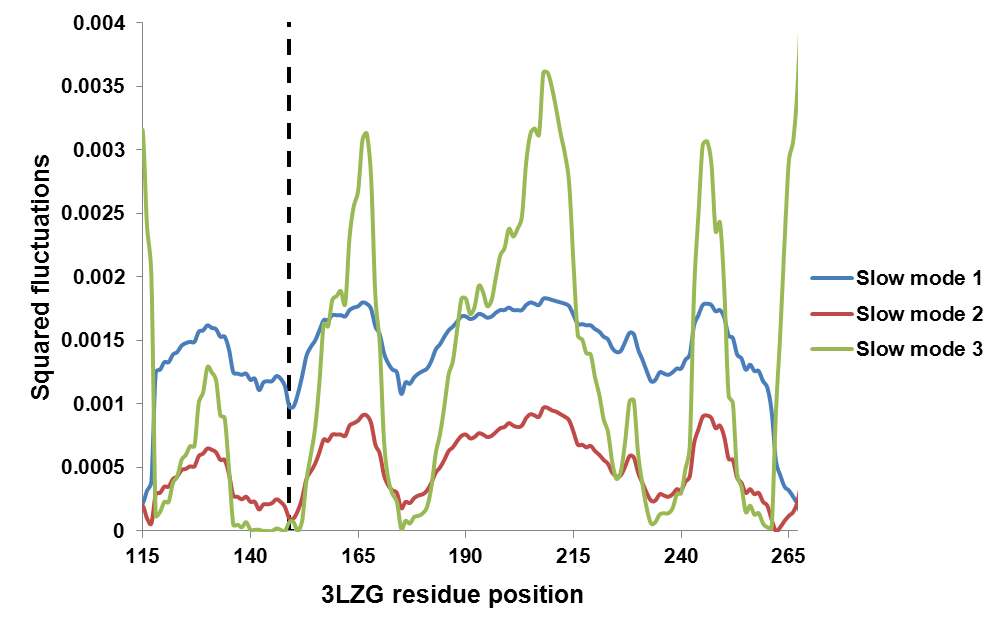
**Fig. S8**


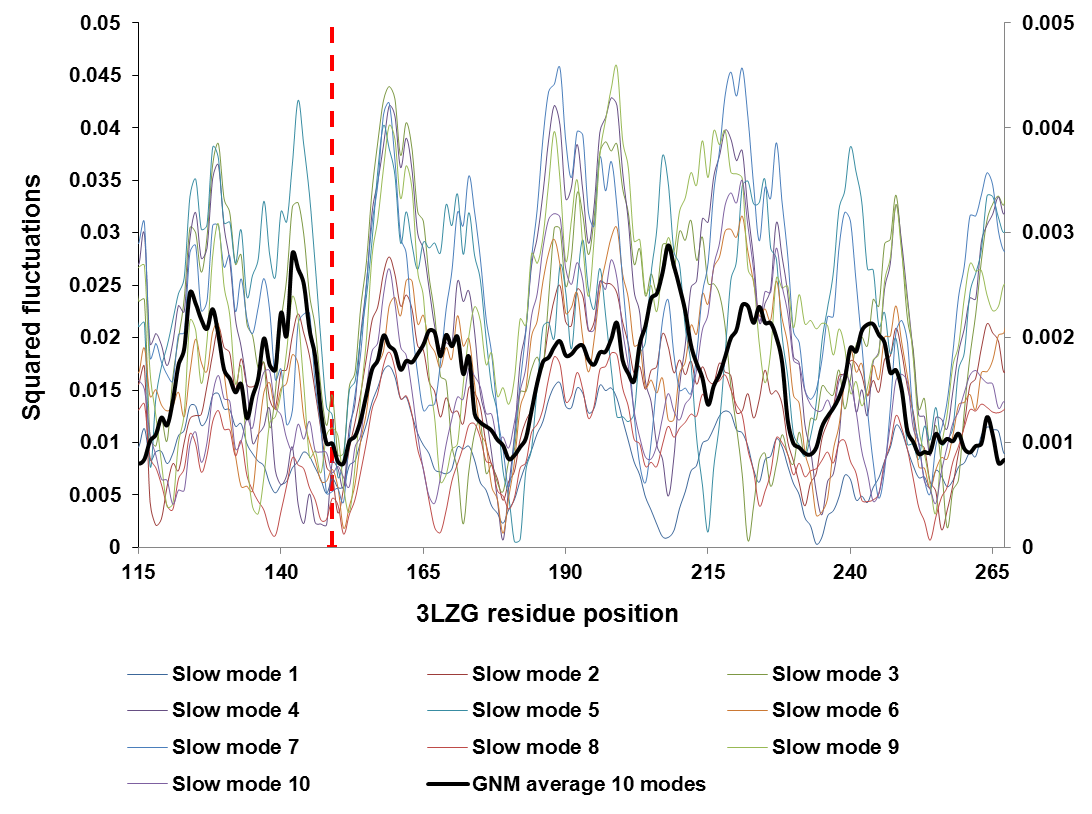
 **Fig. S9**

**Fig. S10**


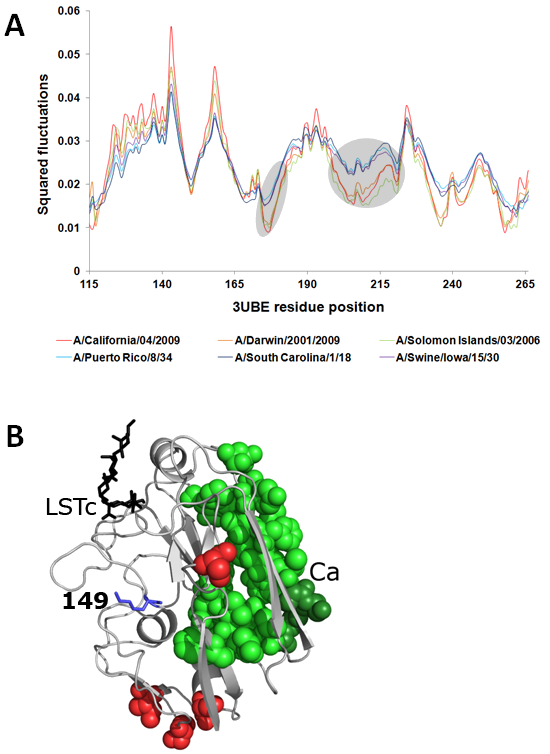

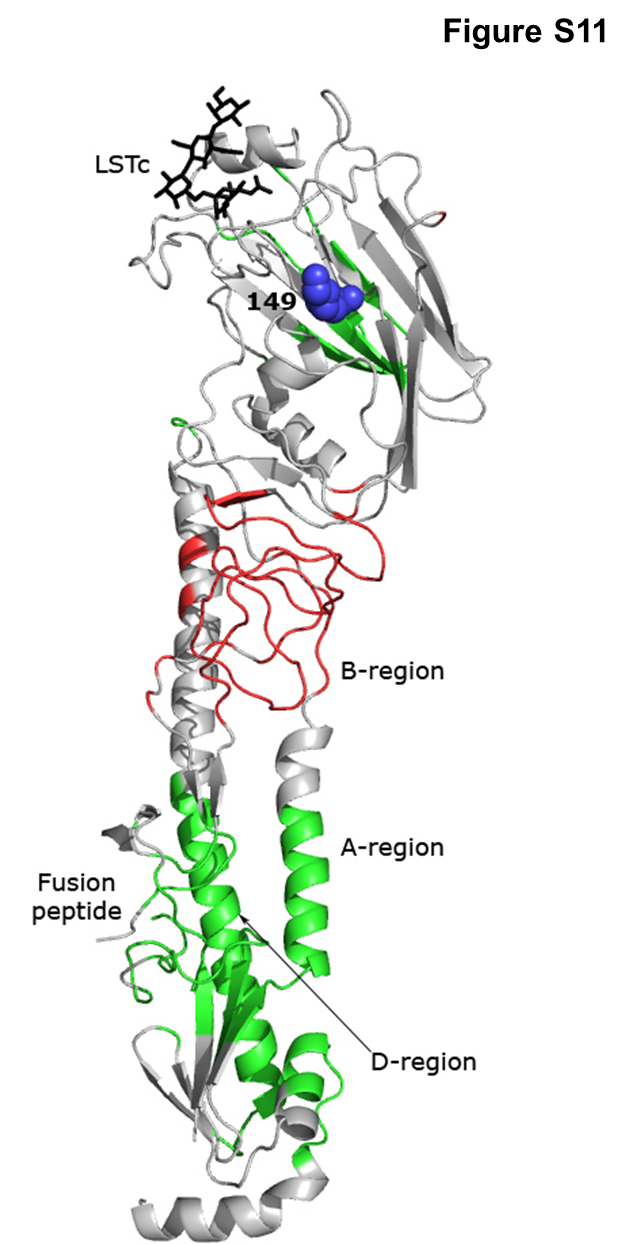

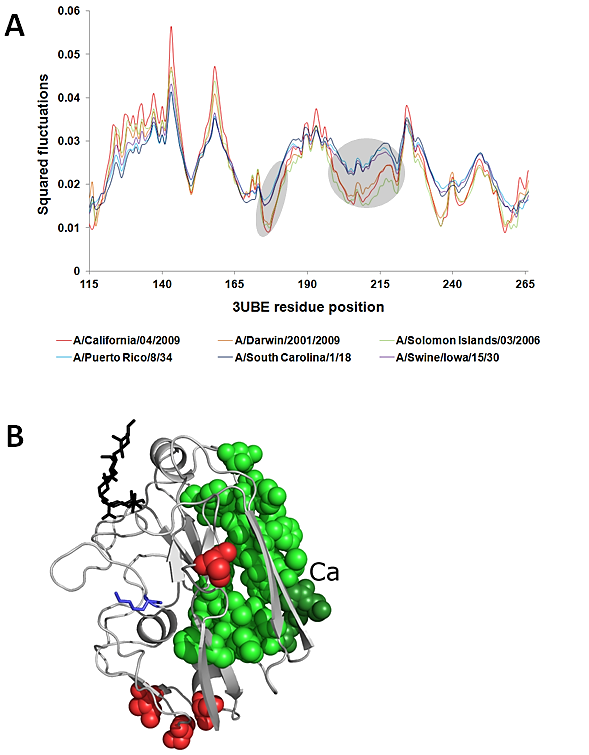


**A**

**B**

**C**

**SI Figure legends**

**Supplementary Fig. S1.** **Virus shedding of TRsw influenza viruses in ferrets in transmission experiments.** Ferrets were inoculated intranasally with 10^6^ EID_50_/ml of NC/02 (*A*), NC/02:TN/09^NA,M^ (*B*), NC/02^HA149^ (*C*), NC/02^HA149^:TN/09^M^ (*D*), NC/02^HA149^:TN/09^NA^ (*E*), and NC/02^HA149^:TN/09^NA,M^ (*F*) influenza viruses. Nasal wash samples were collected on 1, 2, 4, 6, 8, 10, and 12 dpi for inoculated ferrets and on 1, 3, 5, 7, 9 and 11 post-contact ferrets as described in Methods. Virus titers were determined by TCID_50_ assay using MDCK cells and limitation of virus detection was one log_10_TCID_50_/ml (dotted line). These data show the requirement of the EA NA and M gene segments in addition to the HA 149K substitution for airborne transmission. Although individual gene segments and/or substitution affected the degree of direct transmission, none transmitted to airborne contacts.

**Supplementary Fig. S2.** **Virus shedding of NC/02^HA133^ influenza viruses in ferrets in transmission experiments.** Ferrets were inoculated intranasally with 10^6^ EID_50_/ml of NC/02^HA133^ virus. A percentage of transmissibility (*A*) and viral titer (*B*) were determined as described in Fig.1 and Fig. S1. The data show that the HA 133K substitution had no effect on viral transmission.

**Supplementary Fig. S3.** **Attachment of NC/02 and NC/02^HA149^ viruses to ferret nasal turbinates.** The nasal turbinates from normal ferret were incubated with florescence only (*A*), florescence-labeled NC/02 (*B*), and NC/02^HA149^ (*C*) viruses and visualized using a Zeiss LSM 510 Meta confocal microscope. To visualize the cell nuclei, sections were counterstained with DAPI (blue). The representative images of fluorescence-labeled virus attached in ferret nasal turbinate sections are shown (magnification x 40). Consistent with an increased titer in the turbinate’s of NC/02^HA149^ infected animals, the binding of this virus was superior to that of NC/02.

**Supplementary Fig. S4.** **Virus shedding of TN/09 (*A*) and TN/09^HA149^ (*B*) influenza viruses in ferrets in transmission experiments.** Ferrets were inoculated intranasally with 10^6^ EID_50_/ml of virus. Viral titers were determined as described in Fig.1 and Fig. S1.

**Supplementary Fig. S5.** **The NA enzyme kinetics of TN/09, NC/02, NC/02^HA149^, NC/02:TN/09^NA,M^, and NC/02^HA149^:TN/09^NA,M^ viruses using 4-MU-NANA as substrates.** The kinetic determine the Michaelis constant (*KM*) and maximum velocity (*Vmax*) of substrate conversion and as described in Methods. The representative images were performed three independent experiments.

**Supplementary Fig. S6.** **HA residue 149 is centrally located in the receptor binding domain.** Three dimensional structure of A/California/04/2009 (PDB ID: 3UBE). Sidechains forming salt bridges with residue 149 are identified along with LSTc (black) in the receptor binding pocket. Residue K149 is located in the receptor binding sub-domain (green) peripheral to the receptor binding pocket but adjacent to the vestigial esterase sub-domain (yellow), which is positioned atop and stabilizes HA1 (blue) and HA2 (red) chains in the metastable membrane-proximal stalk domain. The backbone of residue 149 forms a hydrogen bond with the backbone of residue G72 in the vestigial esterase subdomain. The residue 149 sidechain forms salt bridges with residues H141 and S146, which are part of a loop proximal to the receptor binding pocket. The x-ray crystal structures of HA-K149 and HA-R149 (present in the structure of PR8, PDB ID: 1RU7) have similar backbone structures. The central location and network of interactions of residue 149 appears to allow it to regulate molecular dynamics and motions in distant parts of the molecule. The three slowest modes of the RBD of A/California/04/2009 (PDB ID: 3UBE). Position 149, marked by the dashed line, appears as a hinge in all three modes. The GNM calculations were conducted using a single HA1 chain, the RBD of which is presented.

**Supplementary Fig. S7. Receptor binding specificity.** HA glycan array analysis (*A*). A list of α2,3- or α2,6-linked glycans used for glycan microarray analysis (*B*). No major differences in binding patterns were observed.

**Supplementary Fig. S8. The three slowest modes of the RBD of A/California/04/2009 (PDB ID: 3UBE).** Position 149, marked by the dashed line, appears as a hinge in all three modes. The GNM calculations were conducted using a single HA1 chain, the RBD of which is presented.

**Supplementary Fig. S9.** **The ten slowest ANM modes resemble the average of the 10 slowest GNM modes.** Most importantly, position 149, marked by the dashed line, appears as a major hinge in each of the 10 slowest GNM and ANM modes. Both the ANM and GNM calculations were conducted using a single HA1 chain of A/California/04/2009 (PDB ID: 3UBE), the RBD of which is presented.

**Supplementary Fig. S10.** **Differences of lysine- vs. arginine-containing variants in the residue fluctuations.** Mode 7 of A/Darwin/2001/2009 (PDB ID: 3M6S), A/California/04/2009 (PDB ID: 3UBE), A/Solomon Islands/03/2006 (PDB ID: 3SM5, A/Berlin/6/2006 for HA2), A/Swine/Iowa/15/30 (PDB ID: 1RVT), A/South Carolina/1/18 (PDB ID: 1RUZ), A/Puerto Rico/8/34 (PDB ID: 1RU7) (*A*). The first three have lysine in position 149 and the last three have arginine. The regions with the main difference between lysine- and arginine-containing variants are highlighted in grey. It is evident from the curves that the R149K mutation consistently affects this mode of motion. The calculations were conducted using the full trimeric HA structures; results are shown only for the RBD. The predicted effect of the R149K mutation on RBD residue fluctuations portrayed on the structure of A/California/04/2009 (PDB ID: 3UBE) (*B*). The RBD is represented in grey cartoon with position 149 in blue sticks. Positions that exhibit decreased (green) or increased (red) fluctuations in variants that have lysine, rather than arginine, in position 149 in mode 7 are marked in atom spheres model representation. The human receptor analogue is shown in black bond-sticks representation. The R149K mutation is predicted to greatly affect the fluctuations of spatially distant regions. The structure of A/California/04/2009 (PDB ID: 3UBE) in grey cartoon representation with position 149 in blue atom spheres model. Positions that exhibit decreased and increased fluctuations in variants that have lysine, rather than arginine, in position 149 in mode 7 are marked in green and red, respectively (*C*). The human receptor analogue is shown in black bond-sticks representation. The figure was made using PyMOL.

**Supplementary Video S1.** **The stretching/compressing motion of ANM mode 7 of the A/California/04/2009 HA (PDB ID: 3UBE).** The HA trimer is presented using cartoon representation, each monomer in a different color, with position 149 highlighted in (red) atom-spheres model. The sialic acid analogue is shown using black bond-sticks representation, and the Cα atoms of the binding site residues are represented as spheres seven frames were included to properly depict the motion. The movie was made using PyMOL.
